# Modeling Effective Dosages in Hormetic Dose-Response Studies

**DOI:** 10.1371/journal.pone.0033432

**Published:** 2012-03-16

**Authors:** Regina G. Belz, Hans-Peter Piepho

**Affiliations:** 1 Agroecology Unit, University of Hohenheim, Institute of Plant Production and Agroecology in the Tropics and Subtropics, Stuttgart, Germany; 2 Bioinformatics Unit, University of Hohenheim, Institute for Crop Science, Stuttgart, Germany; Pennsylvania State University, United States of America

## Abstract

**Background:**

Two hormetic modifications of a monotonically decreasing log-logistic dose-response function are most often used to model stimulatory effects of low dosages of a toxicant in plant biology. As just one of these empirical models is yet properly parameterized to allow inference about quantities of interest, this study contributes the parameterized functions for the second hormetic model and compares the estimates of effective dosages between both models based on 23 hormetic data sets. Based on this, the impact on effective dosage estimations was evaluated, especially in case of a substantially inferior fit by one of the two models.

**Methodology/Principal Findings:**

The data sets evaluated described the hormetic responses of four different test plant species exposed to 15 different chemical stressors in two different experimental dose-response test designs. Out of the 23 data sets, one could not be described by any of the two models, 14 could be better described by one of the two models, and eight could be equally described by both models. In cases of misspecification by any of the two models, the differences between effective dosages estimates (0–1768%) greatly exceeded the differences observed when both models provided a satisfactory fit (0–26%). This suggests that the conclusions drawn depending on the model used may diverge considerably when using an improper hormetic model especially regarding effective dosages quantifying hormesis.

**Conclusions/Significance:**

The study showed that hormetic dose responses can take on many shapes and that this diversity can not be captured by a single model without risking considerable misinterpretation. However, the two empirical models considered in this paper together provide a powerful means to model, prove, and now also to quantify a wide range of hormetic responses by reparameterization. Despite this, they should not be applied uncritically, but after statistical and graphical assessment of their adequacy.

## Introduction

Reports of the phenomenon of stimulatory effects of low dosages of a toxicant, or in fine hormesis, are accumulating in many toxicological sciences and hormetic dose-response data sets appear the rule rather than the exception [1], [2]. Consequently, there has been an increased interest in statistical models that incorporate these effects [2], [3]. Hormesis has been found within all groups of organisms, for a wide range of endpoints, and it is induced by physical or chemical stress factors including many herbicides and other phytotoxins [4], [5], [6]. Although evidence has accumulated that hormesis is a true and reproducible dose-response occurrence, its authenticity has long been questioned and viewed as experimental outlier, especially if only one dose exhibited a response increase. Hence, one major concern of early research in this field was to recognize those cases where hormesis exists and to adequately establish the significance of the phenomenon [2], [3]. Since the recognition of the entire dose-response curve provides the strongest and most reliable basis for testing of hormesis, there was a need for developing robust statistical models describing hormetic effects [2]. Although meanwhile a broad class of mathematical-statistical models exist that allow the incorporation of hormesis and testing its significance, two empirical models gained most credit for modeling hormetic data in dose-response studies with natural or synthetic phytotoxins in plant biology. Both are derivatives of the general monotonic log-logistic function most-used in herbicide dose-response studies [7], [8] and reduce to this in the absence of hormesis [2], [3]. The Brain and Cousens model [9] is one of the earliest and well-known dose-response models allowing for hormesis and the assessment of its significance (Equation (1)). The model can be written as
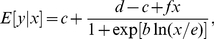
(1)where *c* denotes the response at infinite doses, *d* denotes the mean response of the untreated control, and *f* denotes the rate of stimulation at doses close to zero (*f*>0 as a necessary condition for the presence of hormesis), while parameters *b* and *e* have no straightforward biological meaning [9]. Although the model has been successfully used in several plant studies (e.g., [3], [10], [11], [12], [13]), it has some drawbacks making it not especially robust and flexible when applied to real data [2]. Particularly the fact that the model is confined to values of *b* larger than one can be problematic if data giving curves with inherently low slopes are to be fit [2]. Based on the fact that Cedergreen et al. [2] could not find significant hormesis modelling 51 dose-response data sets of herbicide toxicity with the Brain and Cousens model [9], they modified the equation by replacing *fx* in Equation (1) by 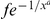 (Equation (2)):
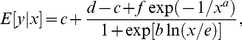
(2)where *f* denotes the theoretical upper bound of the hormetic effect (*f*>0 as a necessary condition for the presence of hormesis), while parameters *a*, *b*, and *e* have no straightforward biological meaning. This new model was found to be much more robust and apt to adequately fit 69% of the 51 dose-response data sets evaluated in Cedergreen et al. [2]. Since its development, the model has been successfully used in several studies on plant hormesis and was shown to feature a higher flexibility to capture variation in hormetic data (e.g., [6], [14], [15], [16], [17]). The only apparent drawback seems to be the fact that the parameter *a* has to be fixed, because there are rarely enough data available to determine the rate of increase statistically [2]. Since their introduction both models have been frequently used in plant biology and have considerably helped to award the phenomenon of hormesis more respectability in this research area by a sound statistical validation. However, as the findings of significant hormesis were growing, the demands of modelling this low-dose phenomenon changed from a mere proof of its evidence to a precise statistical quantification of important hormetic dosages with their standard errors and confidence intervals [2], [3], [10]. Among the quantitative features describing the expression of a hormetic response are the dose where the hormetic effect is maximal (*M*), the response at dose *M* (*y*_max_), the dose where the hormetic effect disappears or the limited dose for stimulation (*LDS*), and the dose causing 50% reduction in mean response of the untreated control (*ED*_50_). Furthermore, the size of the hormetic effect is often described by the dose range of the hormetic zone representing the distance between *M* and *LDS* doses and the distance between *LDS* and *ED*_50_ doses [2], [10], [18]. These values are, however, not directly accessible applying either the Brain and Cousens [9] or the Cedergreen et al. model [2] in its initial forms (Equations (1) and (2)). In order to allow the direct estimation of these dosages, Schabenberger et al. [10] reparameterized the Brain and Cousens model [9] to obtain estimates of *M*, *LDS*, and arbitrary *ED_K_* doses with their standard errors and confidence intervals. Cedergreen et al. [2] applied the delta method and the statistical software *R* with the add-on package *drc* to do so fitting their model to hormetic data. This, however, has hitherto allowed merely estimating *ED_K_* doses with statistical properties and to extract *M* doses but without standard errors or confidence intervals. *LDS* dose estimations are lacking which is why *ED_1_* doses are usually estimated to characterize the transition from stimulation to inhibition (e.g., [2], [14], [15]). Therefore, applications of the Cedergreen et al. model [2] are currently limited to cases where *M* estimations are sufficient without statistical properties and where *LDS* estimations in form of *ED_1_* doses are adequate. This is certainly applicable for most hormesis evaluations, however, not so for example for the prediction of hormesis in mixtures by joint action analysis [11]. To assess the necessary quantities in such cases there is currently no other choice of a properly parameterized function than the less flexible Brain and Cousens model [9] even if the Cedergreen et al. model [2] may fit the data better. The bias potentially incurred by relying on a single model that is known to have serious drawbacks is critical, since conclusions on treatment effects are conditional on the suitability of the model used [3], [10]. However, in order to judge a potential impact of hormetic model selection on effective dose estimates and the conclusions drawn from such evaluations, it is necessary that both functions addressed herein are parameterized properly to allow inference about stimulatory quantities.

The aim of the present work was therefore to provide a general method for reparameterization of the Cedergreen et al. model [2] to allow for the estimation of the effective dosages *M* and *LDS* and arbitrary *ED_K_* doses with their standard errors and confidence intervals. Based on this, the quantification of the hormetic effect by the Cedergreen et al. [2] and the Brain and Cousens model [9] was compared with regard to the aptness of describing various empirical hormetic data sets and the impact on effective dose estimations, especially in case of a substantially inferior fit by one of the two models. For this purpose 23 data sets were evaluated describing the hormetic responses of four different test plant species exposed to 15 different chemical stressors in two different experimental dose-response test designs.

## Methods

### Reparameterization

The estimation of effective dosages with the Brain and Cousens model [9] by reparameterization through defining relationships is described in detail in Schabenberger et al. [10]. Table 1 gives an overview of the respective model expressions. Parameterizations of the Cedergreen et al. model [2] were done by modifying the nonlinear model to contain an *ED_K_* or an *M* dosage by defining a conditional equation that was then plugged in for *d* in case of *ED_K_* and for *f* in case of *M* values. *LDS* values represent the special case of *K* = 0. Upon replacing *x* by the respective target dosage in the remodelled parameter equation, the latter replaced the respective parameter in Equation (2). The target dosage can thus be directly estimated with standard error and confidence interval.

**Table 1:** Parameterizations of the Brain and Cousens model [9] after Schabenberger et al. [10] to estimate particular dosage effects.

**Parameterization for estimating ** ***ED*** **_K_**
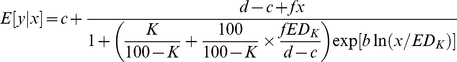
**Parameterization for estimating ** ***LDS*** ** (** ***ED*** **_K = 0_)**
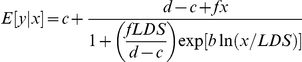
**Parameterization for estimating ** ***M***
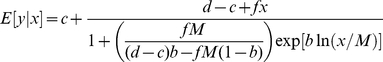

#### Conditional Equation for EDK

To make the Cedergreen et al. model [2] depend on an arbitrary effective dose for *K*% inhibition [(100-*K*)% response] the defining relationship is
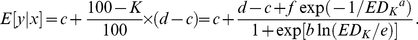
(3)Solving Equation (3) for *d* (Table 2) and substituting into Equation (2) yields a hormetic dose-response model with five parameters (*c*, *b*, *e*, *f* and *ED_K_*) allowing for the estimation of any effective dosage. In terms of *ED*_50_, for example, the model expression corresponds to
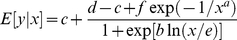
(4)with 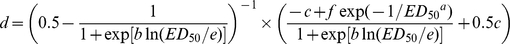.

**Table 2:** Parameterizations of the Cedergreen et al. model [2] to estimate particular dosage effects.

**Parameterization for estimating ** ***ED*** **_K_: parameter to be replaced in ** **Equation (2)**
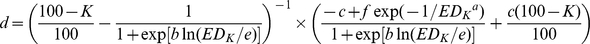
**Parameterization for estimating ** ***LDS*** ** (** ***ED*** **_K = 0_): parameter to be replaced in ** **Equation (2)**
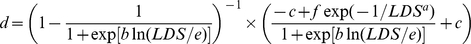
**Parameterization for estimating ** ***M*** **: parameter to be replaced in ** **Equation (2)**


#### Conditional Equation for M

Here, the premise that the first derivative of *f* with respect to *x* must equal zero defines the following relationship
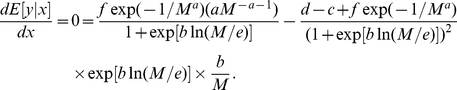
(5)Solving Equation (5) for *f* (Table 2) and substituting into Equation (2) yields a hormetic dose-response model with five parameters (*d*, *c*, *b*, *e*, and *M*). As stated by Schabenberger et al. [10], the reparameterized equations appear complicated at first view, but can be coded easily into a nonlinear regression package (Tables S1 and S2).

### Biological Data

The models were tested on 23 dose-response data sets that were generated using two different bioassay designs. The first set of data resulted from germination bioassays that evaluated the effect of various phytotoxins, their binary mixtures or phytotoxic plant extracts on the root length growth of different test plant species (*Amaranthus hybridus* L., *Lactuca sativa* L., or *Medicago sativa* L.). The second data set consisted of produced root length of *Sinapis alba* L. exposed to root exudates of cereal crops in a hydroponic co-culture.

#### Germination Bioassays

The hormetic effect of two natural isothiocyanates (allyl- and 2-phenylethyl-isothiocyanate; Lancaster Synthesis) was evaluated using *A. hybridus* as test species. *A. hybridus* seeds were exposed for 7 d in Petri dishes in a growth cabinet to aqueous solutions of the isothiocyanates in concentrations ranging from 0–1 µmol/ml under the conditions described in Petersen et al. [19]. *L. sativa* cv. Maikönig (lettuce) was used to test the effect of: (1) natural phytotoxins [parthenin, tetraneurin-A (isolated as described in Belz et al. [20]); 2-amino-3*H*-phenoxazin-3-one (APO; synthesized after Gagliardo and Chilton [21]); *trans*-ferulic acid (Aldrich)], (2) synthetic phytotoxins [2-(*p*-chlorophenoxy)-2-methylpropionic acid (PCIB; Aldrich), glyphosate (Glyfos, Glyfos Supreme (Stähler GmbH); Roundup Speed (Scotts Celaflor)], (3) phytotoxic plant leaf extracts of *Parthenium hysterophorus* L. (produced as described in Belz et al. [20]), or (4) mixtures of these. Either seeds of *L. sativa* or, in case of glyphosate treatments, 2 d-old seedlings pregerminated in methanol (4%) were exposed for 5 d in 6-well cell culture plates in a growth cabinet to aqueous solutions of the phytotoxins (0–148 µmol/ml) or the leaf extract (0–27 mg dry mass/ml). Lettuce assays were performed under the conditions described in Belz et al. [11]. *M. sativa* was used to evaluate the effect of the natural phytotoxin benzoxazolin-2(3*H*)-one (BOA; Fluka). Seeds of *M. sativa* were exposed for 6 d in Petri dishes in a growth cabinet to aqueous BOA solutions in concentrations ranging from 0–4 µmol/ml under the conditions described in Belz et al. [22]. The final root length (≥1 mm) was measured as response variable for all test species.

#### Hydroponic co-culture

The hormetic effect of plant-produced phytotoxins in root exudates of *Triticum aestivum* L. cv. Contra or *Hordeum vulgare* L. cv. Finesse on root length growth of *S. alba* cv. Albatros was evaluated by a co-culture of four pregerminated *S. alba* plants with increasing densities of pregerminated crop plants (0–30 plants/290 ml) in hydroponics. Assays were performed under greenhouse conditions for 5 d under the conditions described in Belz and Hurle [23]. The increase in root length of *S. alba* was measured as response variable.

### Statistical methods

Root length (*y*) as a function of dose (*x*) was fitted to Equations (1) and (2) using IBM SPSS® Statistics (estimation method Levenberg-Marquardt; convergence criterion = 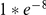). Response variance was stabilized at each dose by using the inverse standard deviation of replicates as weight. All data were fitted to models using a lower limit of zero for *c*. All parameters were freely estimated with the exception of *a* in Equation (2) that was fixed between 0.07 and 1.75 according to the smallest residual sum of squares when hormetic responses could not be described without restrictions on *a* [2]. The range of preset *a* values was broadened as compared to Cedergreen et al. [2] in order to cope with the wide range of hormetic responses in the present data sets. The values of *M*, *LDS*, and *ED*_50_ were estimated by reparameterizations according to Schabenberger et al. [3], [10] for the Brain and Cousens model [9] (Table 1) and by the equations given in Table 2 for the Cedergreen et al. model [2]. Data were first fitted to the original models (Equations (1) and (2)) using starting values for parameters that were graphically deduced from raw data graphs. Parameter *d* was preset according to the mean value of the untreated control, *f* was preset at either 0, 1, 10, or 100 depending on the size of the hormetic response, *a* was also set according to the size of the hormetic response at 0.1, 0.5, or 1, *b* was preset between 1–3 depending on the steepness in the inhibitory dose range, and the starting value for *e* was preset slightly lower than the graphically anticipated *ED*_50_. If the iteration failed, the starting values were adapted so long as all parameters could be properly estimated. These final starting values were subsequently used for reparameterizations of effective doses whose starting values were preset according to the graphically anticipated values. The corresponding response *y*_max_ (absolute and relative to *d*) at *M* was calculated as estimation at *x* = *M* with standard error and confidence interval using SAS®.

#### Significance of hormesis

The significance of hormesis was assessed by the estimation of *f* for both models [2], [10]. According to Schabenberger et al. [10] a hormetic effect is significant at the 0.05 probability level if the 95% confidence interval (CI_95_) for the estimate of *f* does not cover the value zero. With both models either the original models (Equations (1) and (2)) can be used to test for hormesis or their *ED_K_* parameterizations (Tables 1 and 2).

#### Model comparisons

The quality of the description of responses by the regression models was initially assessed by an *F* test for lack-of-fit (*α* = 0.05) and by graphical agreement between observed and fitted values. Model comparisons for best fit were furthermore based on residual sum of squares and residual degrees of freedom (*SS*_res_ */df*_res_) and the Pseudo-*R* ^2^ measure (1-*SS*_res_/*SS*_corrected_) that can be used as a quality parameter for the models used here although with caution [10]. The importance of differences between estimated parameters and effective doses of the two models was judged by the degree of overlap of the CI_95_. As this decision rule does not provide the intended 5% type-I error rate [10], it serves merely as an indication for the likelihood of significant differences. Finally, the relative bias between model estimates was calculated following Schabenberger and Birch [3] as 100*(estimate_2_-estimate_1_)/estimate_1_ where estimate_1_ represents the values derived from the model providing the better fit.

## Results

### Case 1 – both models not suitable

**Figure 1:**
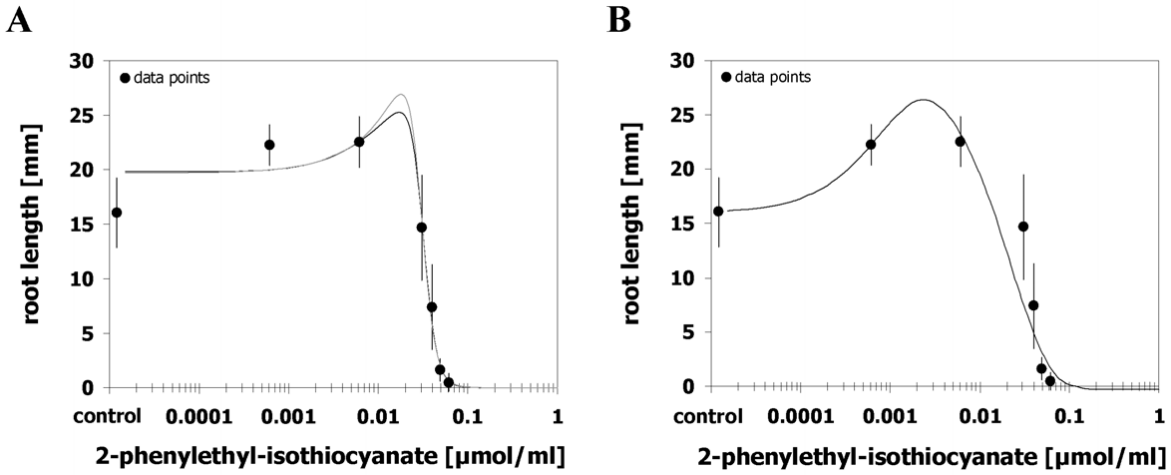
Both logistic hormetic models unsuitable. Dose-response relationship for the effect of 2-phenylethyl-isothiocyanate on root growth of *Amaranthus hybridus* and its description by the hormetic dose-response models after Brain and Cousens [9] (grey curve) or Cedergreen et al. [2] (black curve) (A). (B) Response modelling by the An-Johnson-Lovett Model II [24] (black curve). Only the An-Johnson-Lovett Model II [24] showed a significant hormetic effect. Error bars represent standard deviation.

Out of the 23 hormetic data sets evaluated in this study there was one data set describing the phytotoxic effect of the isothiocyanate 2-phenylethyl that could neither be properly fitted by the Cedergreen et al. model [2] nor the Brain and Cousens model [9] although an analysis of variance with Dunnett's t test (>control; *α* = 0.05) proved two doses as significantly enhanced (Figure 1A). Despite Pseudo-*R* ^2^ values of 0.934 for both models, the graphical agreement between observed and fitted values was poor in each case and the CI_95_ of both *f* values covered zero indicating no significant hormesis (Table S3). The reasons for the inaptness of both models to capture the apparent stimulation may involve the fact of only two hormetic doses and the steepness of the dose-response relationship in the inhibitory dose zone (average *b* = 5.85±0.04). In search of alternative models, the data could be statistically adequately described by the An-Johnson-Lovett Model II [24], a hormesis model based on the ecological-limiting-factor model of Mitcherlich (Figure 1B). Furthermore, modelling with this function proved a significant hormetic effect as the CI_95_ of the two model parameters describing stimulatory responses in this function did not include zero (Table S3). However, although the goodness-of-fit Pseudo-*R* ^2^ value of 0.938 for the An-Johnson-Lovett Model II [24] indicated a satisfactory fit, the graphical comparison between observed values and the fitted curve suggests a risk of overestimating the actual hormetic effect. A more adequate spacing of experimental units within the hormetic dose zone would, however, be necessary to assess this risk.

### Case 2 – Brain and Cousens model more suitable

**Figure 2:**
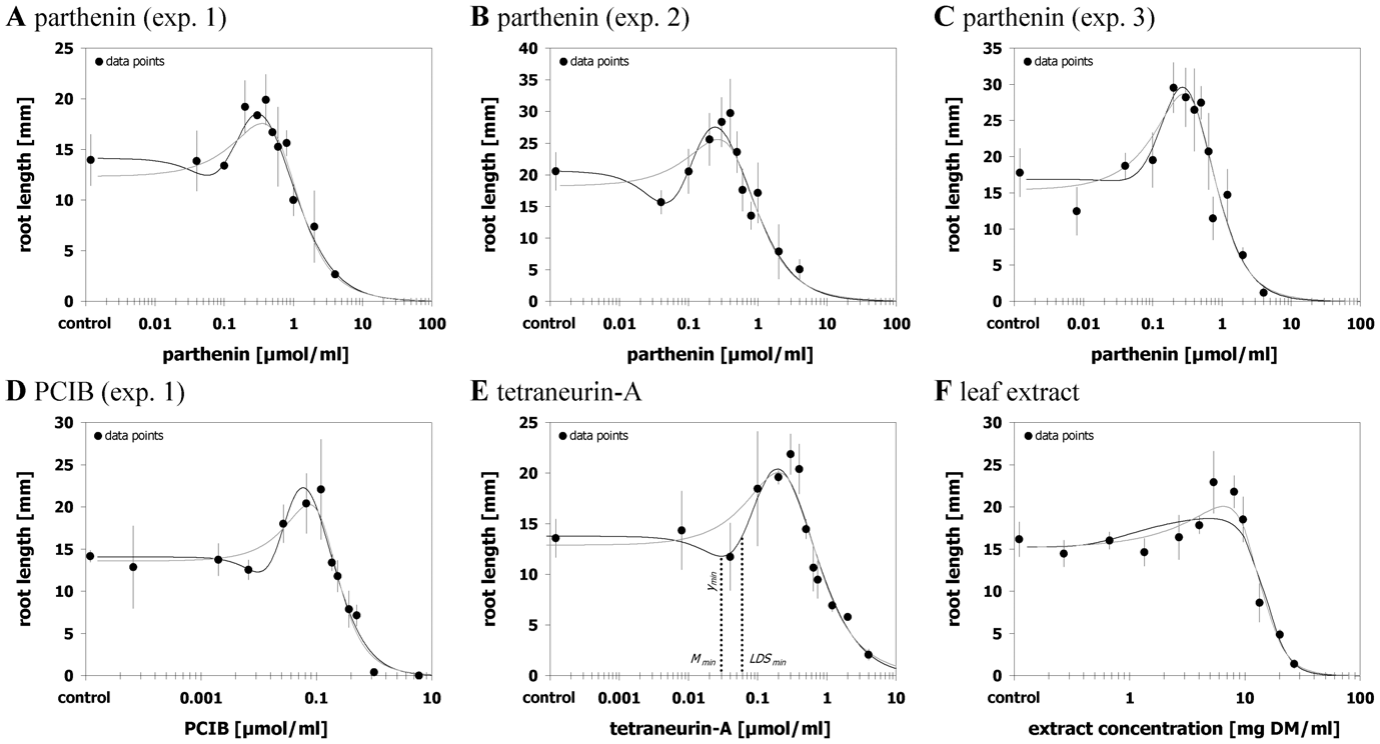
Brain and Cousens model more suitable. Dose-response relationships for effects of different phytotoxins and an aqueous leaf extract of *Parthenium hysterophorus* at the flowering stage on root growth of *Lactuca sativa* and their description by the hormetic dose-response models after Brain and Cousens [9] (grey curve) or Cedergreen et al. [2] (black curve). The Cedergreen et al. model [2] did not detect significant hormetic responses (*f*<0) (A–E) or provided an inferior fit (F). Error bars represent standard deviation. DM = dry leaf mass.

Out of the 23 hormetic data sets there were six obtained with the lettuce assay where the Brain and Cousens model [9] was judged more suitable. At five of these data sets, the Cedergreen et al. model [2] failed to detect significant hormesis in contrast to the Brain and Cousens model [9]. Modelling these five data sets with the Cedergreen et al. model [2] revealed in fact a better goodness-of-fit as judged by lower *SS*_res_ */df*_res_ values and the Pseudo-*R* ^2^ values (0.803–0.943 compared to 0.720–0.920 for the Brain and Cousens model [9]), however, all *f* values were not significantly different from zero either with or without restrictions on the parameter *a* (Figures 2A–E; Table S4). Based on the observed significance of *f* values and the reasonable graphical agreement, we rated the Brain and Cousens model [9] more suitable for these five data sets, however, a more data- and goodness-of-fit-driven judgment may still favour the Cedergreen et al. model [2]. The five data sets comprised all three data sets of parthenin effects, one of five data sets of PCIB effects, and the tetraneurin-A treatment. Modelling the effect of a phytotoxic plant leaf extract revealed a significant hormetic effect with both models; however, this time the goodness-of-fit as well as the graphical agreement was better with the Brain and Cousens model [9] (Figure 2F; Table S4). Thus, out of the 23 data sets evaluated, there was only one where the Cedergreen et al. model [2] clearly provided an inferior fit. Notably, all of these six data sets included at least one data point below the control causing a more or less pronounced drop of the Cedergreen et al. curve [2] for five data sets (Figure 2A–E). With such a drop of the curve, the reparameterized functions for hormetic dosages have more than one solution. Thus, depending on the starting values for effective hormetic doses the initial drop can be quantified by the dose where the drop is maximal (*M*_min_), the response at dose *M*_min_ (*y*_min_), and the dose where the drop disappears and the hormetic effect begins (*LDS*_min_) (Figure 2E). In case of tetraneurin-A for example, a maximum low dose inhibition of 14% was observed at a dose of *M*_min_ = 0.030±0.002 µmol/ml disappearing at *LDS*_min_ = 0.060±0.001 µmol/ml (Figure 2E). Furthermore, the distance between *M*_min_ and *M* comprised a 6.4-fold dose increase and the distance between *LDS*_min_ and *LDS* representing the hormetic dose zone was 10-fold. Hence, the Cedergreen et al. model [2] allows modelling and quantifying this rather unusual feature in hormetic data sets, however, the significance of this low dose toxicity can not be as easily determined as the significance of the stimulatory response by *f* estimation. This would be important, however, in order to judge whether the phenomenon is biologically relevant or just due to experimental variation. Even so, the Cedergreen et al. model [2] proved to be more flexible than the Brain and Cousens function [9] to model hormetic data sets where there is a drop of responses before the initiation of the stimulatory response, just that it failed to indicate the significance of hormesis in such cases.

Comparing the estimates of effective dosages of the two models showed that in all six data sets confidence intervals of *ED*_50_ estimates included the estimate of the other model indicating that differences are not likely to be significant at this response level. In contrast, CI_95_ of hormetic quantities did not overlap between the two models for three of the six data sets, whereby all three curves showed notable variations in *M* estimates, two in *y*_max_, and one in *LDS* values. The magnitude of deviations between both models ranged between 4–25% for *M* and *LDS* estimates and 2–8% for *y*_max_ estimates (Table S5). Thus, while estimates of effective doses in the inhibitory dose range proved widely unaffected if the Cedergreen et al. model [2] failed to significantly model the hormetic effect or provided an inferior fit, quantities characterizing the hormetic effect were more likely impaired.

### Case 3 – Cedergreen et al. model more suitable

**Figure 3:**
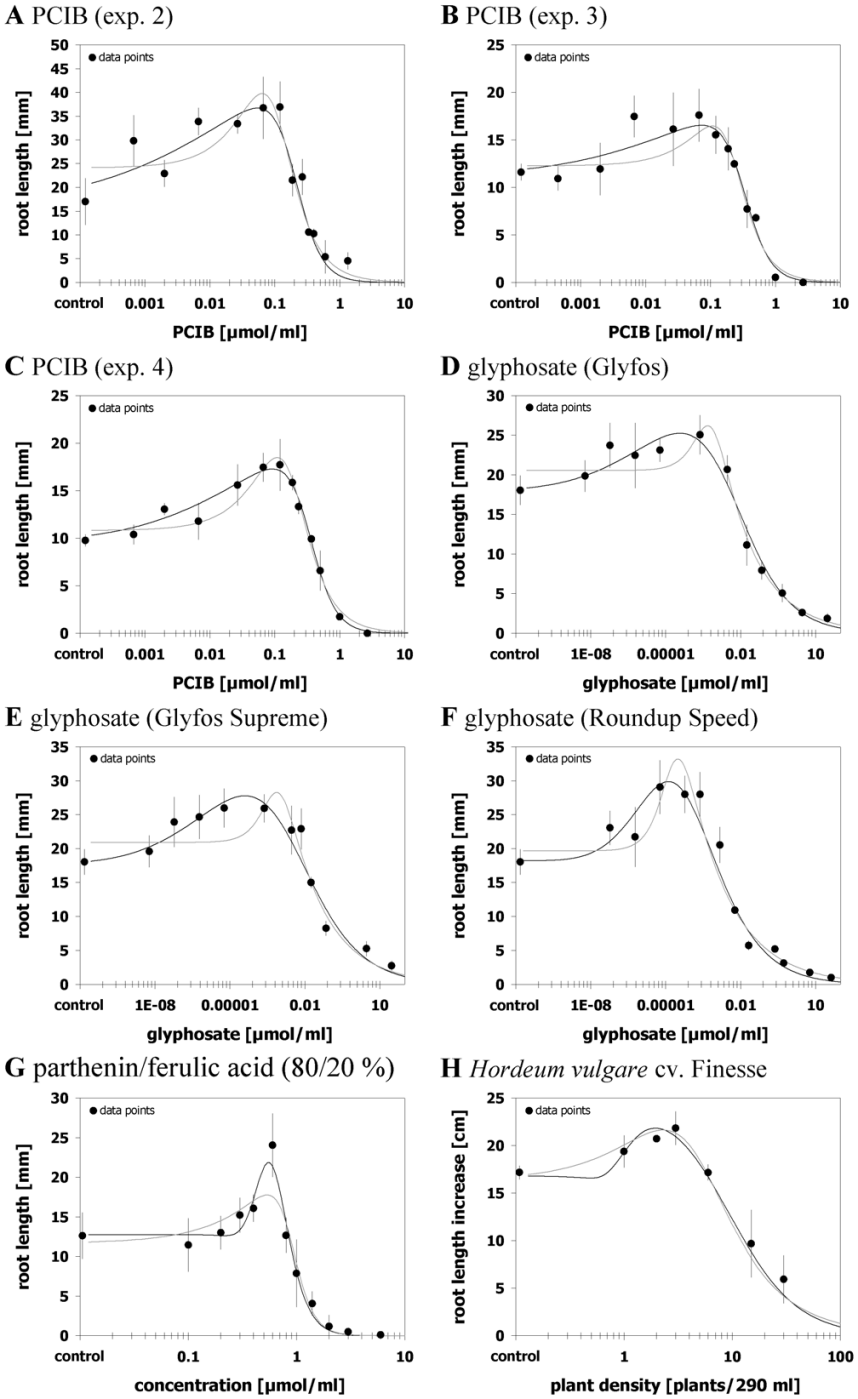
Cedergreen et al. model more suitable. Dose-response relationships for effects of different phytotoxins on root growth of *Lactuca sativa* (A–G) and for effects of root exudates of *Hordeum vulgare* on root growth of *Sinapis alba* (H) and their description by the hormetic dose-response models after Brain and Cousens [9] (grey curve) or Cedergreen et al. [2] (black curve). The Brain and Cousens model [9] detected significant hormetic responses, but provided inferior fits. Error bars represent standard deviation.

Out of the 23 hormetic data sets there were eight where the Brain and Cousens model [9] was less suitable to describe the hormetic response (Figure 3). These eight data sets comprised three of five data sets of PCIB effects in the lettuce assay, all glyphosate treatments, one mixture data set, and one data set reflecting the effect of root exudates of a barley cultivar in the hydroponic bioassay. Although for all these data sets the Brain and Cousens modelling [9] revealed significant hormesis and Pseudo-*R* ^2^ values ranging between 0.874 and 0.969 indicated a satisfactory fit, the graphical agreement between observed and fitted values was partly poor and clearly inferior to the Cedergreen et al. modelling [2]. This was further manifested by consistently lower *SS*_res_/*df*_res_ and each time higher Pseudo-*R* ^2^ values (0.894–0.967) for the Cedergreen et al. modelling [2] (Table S6).

Regarding the shaping of the dose response data that could be better described by the Cedergreen et al. model [2] it is noted that most of these data sets were characterized by an early increase in responses at low doses and a broad hormetic dose range. The average distance between *M* and *LDS* doses as estimated by the Cedergreen et al. function [2] was a 50.3-fold increase in doses as compared to an average of 2.7-fold observed in cases where the Brain and Cousens model [9] provided a better fit (Figure 2).

Comparing the CI_95_ of estimates of effective dosages of the two models showed notable differences at all eight data sets with a considerable bias at all response levels. Non-overlapping CI_95_ between model estimations appeared at four data sets at the *ED*_50_ response level where the Brain and Cousens estimates [9] differed between 5–61% from the values estimated with the Cedergreen et al. model [2]. CI_95_ of *LDS* estimates did not overlap for five data sets and variations ranged between 7–37%. CI_95_ of *M* and *y*_max_ estimates did not overlap for six data sets with the relative bias being most pronounced for *M* values (3–1768%) and least pronounced for *y*_max_ (0–23%) (Table S5). Thus, in cases where the Brain and Cousens model [9] provided a statistically satisfactory but inferior fit as compared to the Cedergreen et al. model [2], effective dosage estimations displayed a severe bias at all response levels, especially at the *M* dose level.

### Case 4 – both models equally suitable

**Figure 4:**
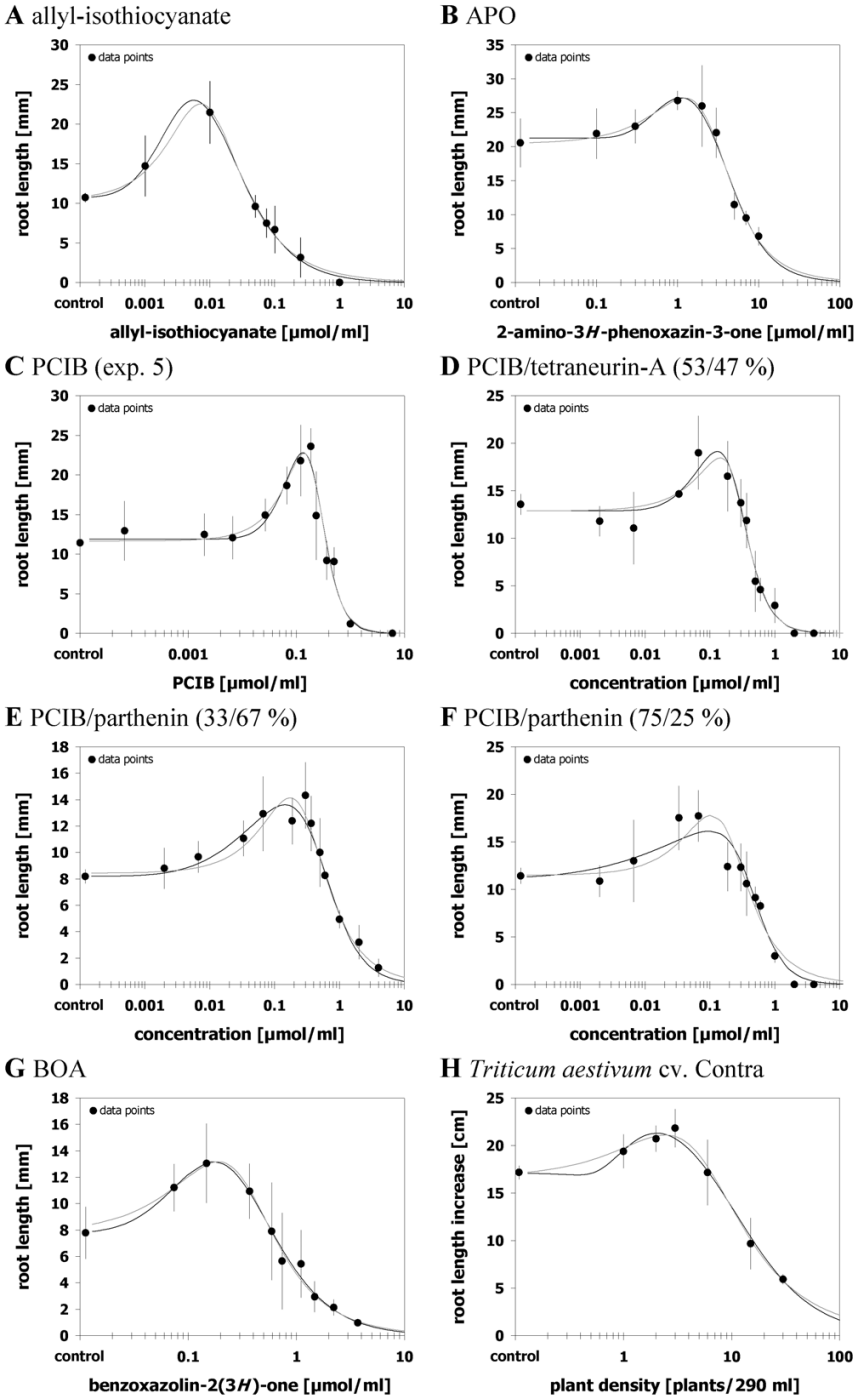
Both logistic hormetic models equally suitable. Dose-response relationships for effects of different phytotoxins on root growth of *Amaranthus hybridus* (A), *Lactuca sativa* (B–F), or *Medicago sativa* (G) and for effects of root exudates of *Triticum aestivum* on root growth of *Sinapis alba* (H) and their description by the hormetic dose-response models after Brain and Cousens [9] (grey curve) or Cedergreen et al. [2] (black curve). Both models provided an adequate, marginally varying fit. Error bars represent standard deviation.

Out of the 23 hormetic data sets there were eight where both models were equally suitable to describe the hormetic response as judged by marginal differences in *SS*_res_/*df*_res_ and Pseudo-*R* ^2^ values and a good graphical agreement between both model fittings. Pseudo-*R* ^2^ values ranged between 0.879 and 0.977. Furthermore, both models yielded a significant hormetic effect for all eight data sets (Figure 4; Table S7). Among these eight data sets were the single effects of allyl-isothiocyanate, APO, and BOA on lettuce, one out of five data sets of PCIB effects, and one data set reflecting the effect of root exudates of a wheat cultivar in the hydroponic bioassay. Furthermore, three data sets describing binary mixture-toxicity effects of phytotoxins that, if applied alone, induce hormesis in the lettuce assay (PCIB, parthenin, and tetraneurin-A) albeit the single effects of the mixture partners proved to be better described by the other model in each case (Figure 4D–F). Thus, joint actions of hormetic compounds showing oppositional model preferences seem to alleviate misspecifications between the two models.

Comparing the estimates of effective doses and *y*_max_ between both models revealed overlapping CI_95_ for all quantities. Notwithstanding, estimated values deviated with the divergence increasing from less than 4% to up to 26% in the order *ED*_50_<*y*_max_<*LDS*<*M* (Table S5). Thus, although unlikely statistically significant, the difference between model estimations was again most pronounced in the hormetic dose range and here especially concerning *M* doses whereby *M* estimates of the Brain and Cousens model [9] always overestimated those obtained by Cedergreen et al. fittings [2]. As a consequence, the estimated hormetic dose zone as expressed as the distance between *M* and *LDS* was consistently lower for the Brain and Cousens modelling [9]. Hence, there are data sets where both models are obviously of the same value regarding a proper description of hormetic responses, but still there appears to be considerable inherent deviation in hormetic quantities that may impair the conclusions drawn depending on the model used.

## Discussion

Results confirm that hormetic dose responses can take on many shapes and that the shaping of the data determines which model fits better [3]. The apparent diversity of hormesis may reflect responses of, e.g., different hormetins, test organisms or species, endpoints, or time periods. This increases requirements with regard to modelling as compared to a less challenging monotonic response. In order to cope with this diversity of hormetic responses, clearly several hormetic candidate models should be evaluated since there is no single best model that is flexible enough to capture the entire variety of shapes and potentially unusual patterns present in the data. However, the need for properly parameterized models allowing inference about the significance of hormesis and quantities of interest [2], [10] narrows the range of currently available functions down to the two models addressed herein. Even so, this study and the study of Cedergreen et al. [2] showed that the two models are able to cover a wide range of hormetic dose responses in plant biology, with the Cedergreen et al. model [2] proving more flexible and able to fit a broader variety of shapes due to the introduction of a second hormetic regression parameter *a*. Nevertheless, the current example of 2-phenylethyl isothiocyanate substantiates that their flexibility with regard to modelling is limited. If the observed inaptness results from the extreme steepness of the curve in the inhibitory dose range and/or a lack of data points within the hormetic dose zone needs to be verified. Cedergreen et al. [2] recommended at least 4–5 hormetic doses to adequately describe and quantify a hormetic response, which is clearly more than the two doses exceeding the control in the current case of 2-phenylethyl isothiocyanate. An adequate spacing of dosages is clearly a prerequisite to reliably model the putative true shape of a hormetic response, nevertheless, modelling hormetic responses with just 1–2 doses exhibiting a response increase is often observed in hormesis research [2], [10], [13], [17].

In cases where the two empirical models addressed herein are inadequate, a range of alternative models are available to describe and prove hormetic responses [3], [24], [25], [26]. In the present case of 2-phenylethyl isothiocyanate, a proper statistical alternative to fit the data was the An-Johnson-Lovett Model II [24]. However, as this empirical model lacks a parameter describing the lower response limit like the *c* value in the Brain and Cousens [9] and the Cedergreen et al. [2] models, introducing an artificial maximum response at a high dose was necessary to avoid a drop of the curve to negative responses at higher doses. Thus, this model requires an adequate spacing of responses in the hormetic and the inhibitory dose ranges. Furthermore, as other alternatives, the model is not parameterized to deduce effective quantities. Hence, in cases where hormetic modelling is forced to switch to alternatives to a Brain and Cousens [9] or a Cedergreen et al. [2] modelling, quantification is intricate and requires advanced statistical skills.

The Cedergreen et al. model [2] constitutes an improved, flexible expansion of the Brain and Cousens model [9] raising the question if the latter is still a competitive model. Cedergreen et al. [2] studied 51 dose-response data sets composed of large, relatively small, and non-hormetic responses. The Cedergreen et al. model [2] adequately fitted 35 of these data sets, while the Brain and Cousens model [9] did not reveal significant hormesis at all. In this study, the Brain and Cousens model [9] detected significant hormesis in 22 out of 23 hormetic data sets while the Cedergreen et al. model [2] did so only 17 times. Thus, there were five cases where only the Brain and Cousens model [9] was able to capture the apparent significance of hormesis. These five cases were all characterized by a more or less pronounced drop of the Cedergreen et al. curve [2] before the initiation of the hormetic effect. According to Cedergreen et al. [2] the drop results if the value of *a* is preset too high. However, the *a* values of the five curves ranged between 0.45 and 0.62 and were, thus, considerably lower as the *a* values of four other Cedergreen et al. curves [2], showing a negligible drop (Figures 3G, 3H, 4B, and 4H; *a* = 0.81–2.50). Thus, the value of *a* may not be decisive for initiating a pronounced drop. Anyhow, results indicate that a pronounced drop of the Cedergreen et al. curve [2] at low doses makes it more difficult to obtain a significant *f* value. On the other hand, this shape of the curve visually better described this pre-hormetic toxicity pattern than the steadily increasing Brain and Cousens model [9] which was unable to capture this phenomenon. The question is, however, if capturing this phenomenon is imperative, i.e., if it is actually biologically relevant and if ignoring it by applying the Brain and Cousens model [9] makes a difference. Evaluating the biological significance of pre-hormetic toxicity is intricate as little information exists on this topic in plant biology and often just one dose below the control value suggests its existence. However, current results confirm that the phenomenon is regularly observed for parthenin, a hormetin showing hormesis as a result of overcompensation of initial inhibitory responses [13], [14]. Furthermore, Sinkkonen et al. [27] observed low doses of toxicants to inhibit the most vigorous individuals within a plant population and claimed that low-dose stimulatory effects are not the only biological phenomenon occurring in the low dose range. As demonstrated, the Cedergreen et al. model [2] may offer a means to quantify this phenomenon. Comparing the CI_95_ for the estimate of *d* with that of *y*_min_ may allow concluding on its significance following the procedure for the significance test for the *f* estimate. Schabenberger et al. [10] pointed out that if parameters are expressed relative to a baseline treatment, a direct estimate of a treatment difference is obtained. According to this, significance of the observed low-dose toxicity would be given in the present study at the 0.05 probability level for two parthenin curves (Figure 2A–B) as here the CI_95_ for the relative estimates of *y*_min_ did not cover the value 100. Nevertheless, much more research effort will be needed to unravel this low-dose toxicity pattern.

Regarding the relative bias incurred by ignoring this low dose toxicity, current results suggest that primarily hormetic quantities may be at risk. However, the magnitude of observed differences between *M*, *LDS*, and *y*_max_ estimates of both models was with 4–25% as distinct as the variations observed in cases where both models provided a similar fit (0–26%). Thus, ignoring this low dose phenomenon did not exceed the magnitude of observed inherent variation between both models. This speaks in favor of a statistically sound Brain and Cousens modeling [9] of hormesis in such cases and reveals the convenience of this model. Additionally, the one data set where the Brain and Cousens model [9] proved superior and the eight data sets where both models proved equally suitable further demonstrates the competitiveness of the model.

For eight data sets the Cedergreen et al. model [2] was statistically and graphically clearly inferior modeling the hormetic effects. Although in each of these cases the use of the Brain and Cousens model [9] was statistically legitimate in terms of lack-of-fit and Pseudo-*R* ^2^, the graphical agreement with the observed data was so poor that substantial deviations from the better fitting Cedergreen et al. curves [2] appeared. As a result, the magnitude of deviations between estimated effective doses was severe and ranged between 3–1768%. Deviations were most pronounced for the three flattish glyphosate curves (139–1758%) confirming the statement of Cedergreen et al. [2] that the Brain and Cousens model [9] can especially cause problems with data describing inherently low slopes. Based on this it is to assume that conclusions drawn from these deviant estimates may considerably diverge at all response levels depending on the model used. This confirms the statement of Schabenberger et al. [10] that assessments of treatment effects are conditional on the correctness of the selected model. Therefore, a sole reliance on statistical measures of lack-of-fit for judging the aptness of a hormetic model can be fatal. Especially the Pseudo-*R* ^2^ measure proved inapt in this regard by pretending a satisfactory fit with values between 0.874 and 0.967 for the poorly fitting Brain and Cousens curves [9]. This statistical measure should therefore only be used as a supplemental criterion. Furthermore, a graphical assessment of the conformity of observed and fitted values is an essential requirement for hormetic model selection.

In cases where both models were statistically and graphically equally suitable to describe the hormetic response, there was still some variation between estimated dosages albeit unlikely significant. If the observed magnitudes of relative bias (0–26%) may influence the assessment of treatment effects may depend on the application and set of empirical data. However, hormetic quantities and especially *M* estimates clearly pose a higher risk in this regard due to the observed higher variability between model predictions as compared to *ED*_50_ dosages. Therefore, in cases where both models offer a suitable fit, a critical question can be how one should objectively decide which model to use.

### Limitations

This study as well as others indicated that the Brain and Cousens model [9] can cause problems when fitting data displaying an early increase in responses at low doses, a broad hormetic dose range, and/or gently sloping curves, while the Cedergreen et al. model [2] only seems to cause problems in case of pre-hormetic toxicity. Furthermore, both models may potentially be limited in case of extremely steep sloping data sets. Cedergreen et al. [2] claimed the necessity to constrain *f* and *b* estimates as a further serious drawback of the Brain and Cousens model [9]. However, in the present study, as in previous applications, *f* and *b* could be easily estimated without restrictions (e.g., [11], [13]). In contrast, there were only six curves in the present study out of the 17 proving significant hormesis with the Cedergreen et al. model [2] were the value of *a* could be estimated without restriction (Tables S2, S4, and S6). Hence, 11 curves required to fix *a* in order to achieve significant *f* estimates. Among these curves were for example all three glyphosate data sets showing 4–6 hormetic doses and, thus, most likely enough data to adequately estimate the size of *a*. Thus, the drawback of restricted parameter estimation seems to apply as well for the Cedergreen et al. model [2]. Hence, both models have real and potential shortcomings raising the question of how competitive they are capturing the diversity of hormesis compared to alternative hormetic models such as the switching functions proposed by Schabenberger and Birch [3] or by Dette et al. [26]. These functions have been rarely addressed for plant hormesis.

Both models now allow the estimation of effective hormetic dosages by properly reparameterized functions. One drawback that seems to remain with this approach is the fact that for each reparameterization, the ensuing model needs to be fitted based on suitable starting values of parameters to ensure convergence of the estimation algorithm [2]. This requires some skill and can be laborious. A further pending question is the competitiveness of reparameterization regarding the ease of model fitting and statistical inference of effective dosages compared to other alternatives like e.g. the *drc* approach [2]. This question may be worth exploring.

Clearly both models and the approach of reparameterization have shortcomings with regard to modeling, however, together they provide a powerful means to model, prove and quantify a wide range of hormetic responses. Despite this, the current approach should not be adopted as a law-of-nature as in some cases other nonlinear models may provide a better fit to the data [3], [10]. Which model best fits observed responses must be statistically and graphically reassessed for every set of empirical data. An uncritical application of a particular model can cause serious misinterpretation especially regarding effective doses quantifying hormesis.

## Supporting Information

Table S1 **Syntax for parameterizations of the Brain and Cousens model** [**9**] **after Schabenberger et al.** [**10**] **.**
(PDF)

Table S2 **Syntax for parameterizations of the Cedergreen et al. model** [**2**] **.**
(PDF)

Table S3 **Regression Parameters for curves displayed in** **Figure 1** **.**
(PDF)

Table S4 **Regression Parameters for curves displayed in** **Figure 2** **.**
(PDF)

Table S5 **Relative bias (%) between effective dosages estimations from curves displayed in** **Figures 2** **,** **3** **, and** **4** **.**
(PDF)

Table S6 **Regression Parameters for curves displayed in** **Figure 3** **.**
(PDF)

Table S7 **Regression Parameters for curves displayed in** **Figure 4** **.**
(PDF)
