# Supplementary material for: Modeling Effective Dosages in Hormetic Dose-Response Studies
Source: PLoS One. 2012 Mar 16;7(3):e33432. doi: 10.1371/journal.pone.0033432 (PMC3306408; doi:10.1371/journal.pone.0033432)
Supplement: Table S1 — Syntax for parameterizations of the Brain and Cousens model [9] after Schabenberger et al. [10] . (PDF) [file pone.0033432.s001.pdf]

Table S1. Syntax for parameterizations of the Brain and Cousens [9] model after Schabenberger et al. [10]. Syntax expression for IBM SPSS® Statistics or SAS® ('ln' replaced by 'log').

***e* estimation**
$E[y\|x]_{Syntax}$ = (*c*+(((*d*-*c*)+(*f***x*))/(1+exp(*b**ln(*x*/*e*)))))
**Parameterization for ED_K estimation**
$E[y\|x]_{Syntax}$ = (*c*+(((*d*-*c*)+(*f***x*))/(1+(((*K*/(100-*K*))+((100/(100-*K*))*(*f***ED*/(*d*-*c*))))*exp(*b**ln(*x*/*ED*))))))
**Parameterization for *LDS* ($ED_{K=0}$) estimation**
$E[y\|x]_{Syntax}$ = (*c*+(((*d*-*c*)+(*f***x*))/(1+(((*f***LDS*/(*d*-*c*)))*exp(*b**ln(*x*/*LDS*))))))
**Parameterization for *M* estimation**
$E[y\|x]_{Syntax}$ = (*c*+((*d*-*c*)+(*f***x*))/(1+(*f***M*/(((*d*-*c*)**b*)-*f***M**(1-*b*)))*exp(*b**ln(*x*/*M*))))
