# Supplementary material for: Modeling Effective Dosages in Hormetic Dose-Response Studies
Source: PLoS One. 2012 Mar 16;7(3):e33432. doi: 10.1371/journal.pone.0033432 (PMC3306408; doi:10.1371/journal.pone.0033432)
Supplement: Table S2 — Syntax for parameterizations of the Cedergreen et al. model [2] . (PDF) [file pone.0033432.s002.pdf]

Table S2. Syntax for parameterizations of the Cedergreen et al. [2] model.
Syntax expression for IBM SPSS® Statistics or SAS® (‘ln’ replaced by ‘log’).

e estimation

$E[y|x]_{Syntax}$ = (c+((d-c)+(f*exp(-1/(x**a))))/(1+exp(b*ln(x/e))))

Parameterization for ED_K estimation

$E[y|x]_{Syntax}$ = (c+(((((((100-K)/100)-(1/(1+exp(b*ln(ED/e)))))**(-1))*(((-c+(f*exp(-1/(ED**a))))/(1+exp(b*ln(ED/e))))+(c*(100-K)/100)))-c)+(f*exp(-1/(x**a))))/(1+exp(b*ln(x/e))))

Parameterization for LDS ($ED_{K=0}$) estimation

$E[y|x]_{Syntax}$ = (c+(((((1-(1/(1+exp(b*ln(LDS/e)))))**(-1))*(((-c+(f*exp(-1/(LDS**a))))/(1+exp(b*ln(LDS/e))))+c))-c)+(f*exp(-1/(x**a))))/(1+exp(b*ln(x/e))))

Parameterization for M estimation

$E[y|x]_{Syntax}$ = (c+((d-c)+(((((exp(-1/(M**a))*(a*(M**(-a-1)))*(1+exp(b*ln(M/e))))-(exp(-1/(M**a))*exp(b*ln(M/e))*(b/M)))**(-1))*((d-c)*exp(b*ln(M/e))*(b/M)))*exp(-1/(x**a))))/(1+exp(b*ln(x/e))))
