# Supplementary material for: Modeling Effective Dosages in Hormetic Dose-Response Studies
Source: PLoS One. 2012 Mar 16;7(3):e33432. doi: 10.1371/journal.pone.0033432 (PMC3306408; doi:10.1371/journal.pone.0033432)
Supplement: Table S3 — Regression Parameters for curves displayed in Figure 1 . (PDF) [file pone.0033432.s003.pdf]

Table S3. Regression Parameters for curves displayed in Figure 1. Parameters from the Brain and Cousens [9] model, the Cedergreen et al. [2] model, the non-hormetic log-logistic model (*f* and *a* equal to zero), and the An-Johnson-Lovett Model II (Liu et al. [24]) fitted to root length data of *Amaranthus hybridus* exposed to 2-phenylethyl-isothiocyanate (Figure 1). Data displayed as mean ± standard error.

Model	*d* [mm]	*f*	*a*	*b*	*e*	Pseudo-R^2	$\frac{SS_{res}}{df_{res}}$
Brain and Cousens model	19.7±0.9	488.3±251.1[ns]	–	5.8±0.8	0.029±0.002	0.934	3.63
Cedergreen *et al.* model	19.8±0.9	98.4±53.7[ns]	0.25[1]	5.8±0.9	0.031±0.002	0.934	3.65
Log-logistic model	20.7±0.7	–	–	6.9±1.3	0.035±0.001	0.929	3.84
**Model**	P_0	S_{max}	K_S	I_{max}	K_I	**Pseudo-R^2**	$\frac{SS_{res}}{df_{res}}$
An-Johnson-Lovett Model II[2]	15.9±1.6	14.7±2.8*	1091.1±392.1*	30.9±2.2	44.8±4.8	0.938	4.76

ns=not significant different from zero; *significant different from zero; [1]fixed; [2] $E[y|x] = P_0 + S_{\max}(1 - e^{-K_S x}) - I_{\max}(1 - e^{-K_I x})$ where P_0 is the control, S_{max} is the maximum stimulatory response, K_S is a constant that describes the response of stimulation to increments of the limiting factor, and I_{max} and K_I are the respective parameters of the inhibitory response; Pseudo-R^2=1-SS_{res}/SS_{corr}; *SS*=residual or corrected sum of squares; *df*=degrees of freedom.
