# Supplementary material for: Modeling Effective Dosages in Hormetic Dose-Response Studies
Source: PLoS One. 2012 Mar 16;7(3):e33432. doi: 10.1371/journal.pone.0033432 (PMC3306408; doi:10.1371/journal.pone.0033432)
Supplement: Table S4 — Regression Parameters for curves displayed in Figure 2 . (PDF) [file pone.0033432.s004.pdf]

Table S4. Regression parameters for curves displayed in Figure 2. Parameters from the Brain and Cousens [9] model (M1) and the Cedergreen et al. [2] model (M2) fitted to root length data of *Lactuca sativa* exposed to various phytotoxins (Figure 2). Data displayed as mean ± standard error.

Figure		*d* [mm]	*f*	*a*	*b*	ED_{50} [µmol/ml]	*M* [µmol/ml]	*LDS* [µmol/ml]	y_{max} [mm]	y_{max} [%]	Pseudo-R^2	$\frac{SS_{res}}{df_{res}}$
2A	M1	**12.3±0.8**	28.0±7.7*	–	**2.1±0.1**	1.904±0.221	**0.353±0.032**	**0.940±0.081**	**17.6±0.4**	143±10	0.920	3.30
	M2	**14.1±0.8**	398.7±215.3 ns	0.62 [1]	**1.4±0.1**	1.718±0.119	**0.304±0.017**	**0.720±0.073**	**18.5±0.4**	131±7	0.943	2.35
2B	M1	**18.2±1.2**	55.0±24.4*	–	**2.0±0.2**	1.560±0.269	0.264±0.046	0.726±0.092	25.6±1.4	140±13	0.720	5.63
	M2	**20.6±1.2**	1774.1±885.9 ns	0.59 [1]	**1.4±0.1**	1.414±0.146	0.240±0.016	0.589±0.071	27.5±1.3	134±10	0.803	3.98
2C	M1	15.4±1.2	82.6±20.8*	–	**2.3±0.2**	1.539±0.192	0.284±0.032	0.866±0.081	28.7±1.3	186±18	0.873	4.97
	M2	16.9±1.0	358.4±193.8 ns	0.58 [1]	**1.8±0.2**	1.464±0.154	0.269±0.030	0.793±0.070	29.6±1.4	175±14	0.877	4.79
2D	M1	13.6±0.5	164.4±48.2*	–	**2.2±0.2**	0.391±0.034	**0.075±0.009**	0.202±0.014	**20.0±1.2**	147±11	0.911	2.62
	M2	14.1±0.5	5206.3±3471.1 ns	0.45 [1]	**1.7±0.1**	0.400±0.027	**0.059±0.004**	0.184±0.014	**22.3±1.0**	159±9	0.923	2.26
2E	M1	12.9±0.9	74.0±20.2*	–	**2.0±0.1**	1.406±0.148	0.199±0.025	0.638±0.068	20.1±0.7	156±12	0.911	3.34
	M2	13.8±0.9	983.3±952.7 ns	0.54 [1]	**1.4±0.1**	1.348±0.118	0.192±0.016	0.577±0.070	20.4±0.7	148±10	0.919	3.07
2F	M1	15.1±0.4	1.0±0.2*	–	4.1±0.3	16.043±0.472	**6.526±0.429**	11.310±0.494	**20.1±0.6**	**133±6**	0.919	2.82
	M2	15.2±0.5	4.2±1.9*	1.13	4.3±0.6	16.504±0.584	**4.910±0.848**	10.897±0.840	**18.6±0.5**	**122±5**	0.901	3.51

ns=not significant or *=significant different from zero; [1]fixed; Pseudo-R^2=1-SS_{res}/SS_{corr}; *SS*=residual or corrected sum of squares; *df*=degrees of freedom; bold characters indicate non-overlapping of 95% confidence intervals of the estimates of both models (except for *f* and *a*).
