# Supplementary material for: Modeling Effective Dosages in Hormetic Dose-Response Studies
Source: PLoS One. 2012 Mar 16;7(3):e33432. doi: 10.1371/journal.pone.0033432 (PMC3306408; doi:10.1371/journal.pone.0033432)
Supplement: Table S5 — Relative bias (%) between effective dosages estimations from curves displayed in Figures 2 , 3 , and 4 . (PDF) [file pone.0033432.s005.pdf]

Table S5. Relative bias (%)[1] between effective dosage estimations from curves displayed in Figures 2-4.

Figure 2	ED_{50}	*M*	*LDS*	y_{max} absolut	y_{max} relativ
A	-9.8	**-13.9**	**-23.4**	**5.1**	-8.4
B	-9.4	-9.1	-18.9	7.4	-4.3
C	-4.9	-5.3	-8.4	3.1	-5.9
D	2.3	**-21.3**	-8.9	**9.9**	8.2
E	-4.1	-3.5	-9.6	1.5	-5.1
F	2.9	**-24.8**	-3.7	**-7.5**	**-8.3**
**Figure 3**	ED_{50}	*M*	*LDS*	y_{max} absolut	y_{max} relativ
A	**-19.1**	18.5	**-30.1**	8.4	**-23.3**
B	-8.9	**46.6**	-10.6	0.0	-7.6
C	-6.6	**18.5**	**-14.8**	**6.9**	-3.4
D	**-55.6**	**1202.7**	-29.8	4.0	**-8.6**
E	**-60.6**	**1767.5**	**-36.3**	0.7	**-14.6**
F	-30.8	**138.5**	**-37.2**	**11.0**	2.4
G	**8.0**	-3.1	**6.9**	**-18.7**	-10.5
H	-4.5	**15.0**	6.8	-0.9	1.5
**Figure 4**	ED_{50}	*M*	*LDS*	y_{max} absolut	y_{max} relativ
A	4.3	16.7	2.3	-1.7	-1.4
B	2.6	25.5	9.6	0.0	3.9
C	-0.2	-4.3	-0.8	0.4	-1.5
D	-0.8	10.8	2.0	-3.6	-3.4
E	-0.2	-16.2	6.1	-3.5	-1.2
F	-4.3	7.1	-13.4	9.9	7.6
G	-3.6	9.2	-0.5	0.0	0.6
H	0.5	-16.7	-11.6	0.9	-2.4

[1]100*($estimate_2$-$estimate_1$)/$estimate_1$ where $estimate_1$ represents the model with the better fit; bold characters indicate non-overlapping of 95% confidence intervals of the estimates of both models
