# Supplementary material for: Modeling Effective Dosages in Hormetic Dose-Response Studies
Source: PLoS One. 2012 Mar 16;7(3):e33432. doi: 10.1371/journal.pone.0033432 (PMC3306408; doi:10.1371/journal.pone.0033432)
Supplement: Table S6 — Regression Parameters for curves displayed in Figure 3 . (PDF) [file pone.0033432.s006.pdf]

Table S6. Regression parameters for curves displayed in Figure 3. Parameters from the Brain and Cousens [9] model (M1) and the Cedergreen et al. [2] model (M2) fitted to root length data of *Lactuca sativa* or *Sinapis alba* exposed to various phytotoxins (Figure 3). Data displayed as mean ± standard error.

Figure		*d* [mm]	*f*	*a*	*b*	ED_{50} [µmol/ml]	*M* [µmol/ml]	*LDS* [µmol/ml]	y_{max} [mm]	y_{max} [%]	Pseudo-R^2	$\frac{SS_{res}}{df_{res}}$
3A	M1	**24.1±1.7**	433.3±125.0*	-	2.3±0.2	**0.326±0.023**	0.064±0.009	**0.179±0.016**	39.8±2.3	**165±16**	0.874	7.05
	M2	**17.1±3.2**	93.5±21.0*	0.13	2.2±0.3	**0.403±0.047**	0.054±0.010	**0.256±0.039**	36.7±1.7	**215±38**	0.894	6.07
3B	M1	12.3±0.6	66.7±26.8*	-	2.4±0.2	0.449±0.028	**0.107±0.011**	0.244±0.017	16.5±1.5	134±15	0.923	3.57
	M2	11.4±0.6	25.4±8.3*	0.15	2.2±0.3	0.493±0.030	**0.073±0.011**	0.273±0.023	16.5±1.0	145±12	0.945	2.61
3C	M1	**10.8±0.3**	124.5±22.5*	-	2.3±0.1	0.556±0.035	**0.109±0.008**	**0.310±0.014**	**18.5±0.7**	171±10	0.943	2.14
	M2	**9.8±0.4**	36.7±4.8*	0.16	2.3±0.2	0.595±0.030	**0.092±0.008**	**0.364±0.017**	**17.3±0.5**	177±5	0.967	1.28
3D	M1	**20.6±0.6**	52653.7±23591.0*	-	**1.3±0.0**	**0.032±0.007**	**0.482±0.128**[2]	2.717±0.674[2]	26.3±1.0	**128±5**	0.969	2.60
	M2	**18.0±0.8**	82.3±17.3*	0.07[1]	**0.4±0.0**	**0.072±0.016**	**0.037±0.015**[2]	3.869±1.308[2]	25.3±0.8	**140±8**	0.974	2.18
3E	M1	**20.9±0.8**	45220.0±21347.3*	-	**1.3±0.0**	**0.065±0.017**	**0.747±0.212**[2]	**5.332±1.384**[2]	28.0±1.4	**134±8**	0.948	4.39
	M2	**17.7±0.9**	126.2±27.6*	0.07[1]	**0.4±0.0**	**0.165±0.051**	**0.040±0.016**[2]	**8.367±2.821**[2]	27.8±1.0	**157±11**	0.963	3.15
3F	M1	19.7±0.9	2171006.6±901463.1*	-	**1.3±0.0**	0.009±0.002	**0.031±0.009**[2]	**0.588±0.139**[2]	**33.2±1.2**	168±9	0.956	4.23
	M2	18.2±1.1	621.0±210.2*	0.10[1]	**0.5±0.0**	0.013±0.003	**0.013±0.005**[2]	**0.936±0.282**[2]	**29.9±1.4**	164±8	0.957	4.14
3G	M1	**11.6±0.6**	14.4±2.8*	-	4.8±0.6	**1.167±0.054**	0.535±0.037	**0.901±0.038**	**17.8±0.7**	153±11	0.933	3.12
	M2	**12.8±0.4**	307.0±135.5*	1.39[1]	4.5±0.4	**1.081±0.040**	0.552±0.020	**0.843±0.023**	**21.9±1.0**	171±10	0.946	2.54
3H	M1	16.3±0.8	4.8±1.3*	-	**1.9±0.2**	15.0±2.2	**2.30±0.29**	6.3±0.7	21.6±0.4	132±7	0.967	2.26
	M2	16.8±0.6	9.1±2.2*	2.50[1]	**1.4±0.3**	15.7±1.9	**1.96±0.12**	5.9±0.7	21.8±0.4	130±5	0.974	1.78

ns=not significant or *=significant different from zero; [1]fixed; [2]in nmol/ml; Pseudo-R^2=1-SS_{res}/SS_{corr}; *SS*=residual or corrected sum of squares; *df*=degrees of freedom; bold characters indicate non-overlapping of 95% confidence intervals of the estimates of both models (except for *f* and *a*).
