# Supplementary material for: Modeling Effective Dosages in Hormetic Dose-Response Studies
Source: PLoS One. 2012 Mar 16;7(3):e33432. doi: 10.1371/journal.pone.0033432 (PMC3306408; doi:10.1371/journal.pone.0033432)
Supplement: Table S7 — Regression Parameters for curves displayed in Figure 4 . (PDF) [file pone.0033432.s007.pdf]

Table S7. Regression parameters for curves displayed in Figure 4. Parameters from the Brain and Cousens [9] model (M1) and the Cedergreen et al. [2] model (M2) fitted to root length data of *Lactuca sativa*, *Amaranthus hybridus* or *Medicago sativa* exposed to various phytotoxins (Figure 4). Data displayed as mean ± standard error.

Figure		*d* [mm]	*f*	*a*	*b*	ED_{50} [µmol/ml]	*M* [µmol/ml]	*LDS* [µmol/ml]	y_{max} [mm]	y_{max} [%]	Pseudo-R^2	$\frac{SS_{res}}{df_{res}}$
4A	M1	10.4±0.5	3974.9±1432.0*	-	**1.7±0.1**	0.120±0.017	0.007±0.002	0.045±0.004	22.6±0.9	211±9	0.902	2.15
	M2	10.8±0.4	1814.6±831.2*	0.25[1]	**1.2±0.1**	0.115±0.012	0.006±0.001	0.044±0.004	23.0±1.3	214±16	0.907	2.05
4B	M1	20.4±1.2	10.5±3.6*	-	**2.1±0.2**	6.440±0.509	1.217±0.175	3.067±0.352	27.2±0.9	133±9	0.927	2.58
	M2	21.3±1.1	28.1±13.0*	0.81[1]	**1.6±0.3**	6.278±0.447	0.970±0.179	2.799±0.390	27.2±1.0	128±8	0.923	2.71
4C	M1	11.6±0.6	122.8±23.6*	-	2.8±0.3	0.541±0.046	0.139±0.013	0.354±0.022	22.7±1.0	195±14	0.910	2.61
	M2	11.9±0.6	156.1±34.2*	0.40[1]	2.5±0.3	0.540±0.045	0.133±0.013	0.351±0.022	22.8±1.0	192±13	0.909	2.64
4D	M1	12.9±0.5	60.6±18.5*	-	2.8±0.3	0.512±0.033	0.144±0.016	0.313±0.022	18.4±1.1	143±12	0.933	2.03
	M2	12.9±0.5	93.9±29.1*	0.39[1]	2.3±0.3	0.516±0.033	0.130±0.014	0.307±0.021	19.1±1.8	148±15	0.935	1.96
4E	M1	8.4±0.3	66.8±16.5*	-	**2.0±0.1**	1.268±0.127	0.173±0.019	0.592±0.036	14.1±0.6	167±10	0.889	1.44
	M2	8.2±0.5	38.6±16.1*	0.26	**1.6±0.2**	1.266±0.123	0.145±0.019	0.628±0.058	13.6±0.6	165±10	0.891	1.45
4F	M1	11.5±0.6	122.0±41.3*	-	2.0±0.1	0.716±0.068	0.105±0.017	0.330±0.034	17.8±1.4	155±14	0.879	2.85
	M2	11.2±0.8	27.4±11.7*	0.19	1.9±0.3	0.748±0.063	0.098±0.021	0.381±0.049	16.2±1.2	144±13	0.905	2.29
4G	M1	7.7±0.8	54.8±15.7*	-	**2.1±0.1**	1.193±0.141	0.189±0.022	0.603±0.083	13.2±0.7	170±22	0.897	1.91
	M2	7.8±0.8	106.0±39.4*	0.43[1]	**1.5±0.1**	1.238±0.144	0.173±0.021	0.606±0.090	13.2±0.7	169±21	0.898	1.89
4H	M1	16.7±0.6	4.2±1.3*	-	**1.8±0.1**	19.1±1.6	2.43±0.40	6.9±0.9	21.1±0.5	127±6	0.977	1.40
	M2	17.2±0.6	9.5±3.8*	1.75[1]	**1.2±0.2**	19.2±1.7	2.03±0.20	6.1±1.1	21.3±0.6	124±5	0.976	1.43

ns=not significant or *=significant different from zero; [1]fixed; Pseudo-R^2=1-SS_{res}/SS_{corr}; *SS*=residual or corrected sum of squares; *df*=degrees of freedom; bold characters indicate non-overlapping of 95% confidence intervals of the estimates of both models (except for *f* and *a*).
